# Modeling the Health and Economic Burden of Hepatitis C Virus in Switzerland

**DOI:** 10.1371/journal.pone.0125214

**Published:** 2015-06-24

**Authors:** Beat Müllhaupt, Philip Bruggmann, Florian Bihl, Sarah Blach, Daniel Lavanchy, Homie Razavi, David Semela, Francesco Negro

**Affiliations:** 1 Swiss HPB (Hepato-Pancreato-Biliary) Center and Department of Gastroenterology and Hepatology, University Hospital Zürich, Zürich, Switzerland; 2 Arud Centres for Addiction Medicine, Zürich, Switzerland; 3 Gastroenterology Department, Ospedale Cantonale, Bellinzona, Switzerland; 4 Center for Disease Analysis (CDA), Louisville, Colorado, United States of America; 5 Consultant, Ruelle des Chataigniers 1, CH-1026 Denges VD, Switzerland; 6 Division of Gastroenterology & Hepatology, Cantonal Hospital St. Gallen, St. Gallen, Switzerland; 7 Divisions of Gastroenterology and Hepatology and of Clinical Pathology, University Hospital, Genève, Switzerland; National Taiwan University Hospital, TAIWAN

## Abstract

**Background:**

Chronic hepatitis C virus infection is a major cause of liver disease in Switzerland and carries a significant cost burden. Currently, only conservative strategies are in place to mitigate the burden of hepatitis C in Switzerland. This study expands on previously described modeling efforts to explore the impact of: no treatment, and treatment to reduce HCC and mortality. Furthermore, the costs associated with untreated HCV were modeled.

**Methods:**

Hepatitis C disease progression and mortality were modeled. Baseline historical assumptions were collected from the literature and expert interviews and strategies were developed to show the impact of different levels of intervention (improved drug cure rates, treatment and diagnosis) until 2030.

**Results:**

Under the historical standard of care, the number of advanced stage cases was projected to increase until 2030, at which point the annual economic burden of untreated viremic infections was projected to reach €96.8 (95% Uncertainty Interval: €36 – €232) million. Scenarios to reduce HCV liver-related mortality by 90% by 2030 required treatment of 4,190 ≥F2 or 3,200 ≥F3 patients annually by 2018 using antivirals with a 95% efficacy rate. Delaying the implementation of these scenarios by 2 or 5 years reduced the impact on mortality to 75% and 57%, respectively.

**Conclusions:**

With today’s treatment efficacy and uptake rates, hepatitis C disease burden is expected to increase through 2030. A substantial reduction in disease burden can be achieved by means of both higher efficacy drugs and increased treatment uptake. However, these efforts cannot be undertaken without a simultaneous effort to diagnose more infections.

## Introduction

The World Health Organization calls hepatitis C virus (HCV) a silent disease [1]. This is not only due to its symptomless clinical course, even when it has progressed to advanced liver disease, but also to the frequency with which it is overlooked and under-funded in the political sphere [2]. HCV infection takes a chronic course in about 80% of those infected, posing a substantial risk of progressing to cirrhosis and hepatocellular carcinoma (HCC) [3–5]. Although HCV incidence and prevalence are decreasing, morbidity and mortality will increase in the next years as complications of end stage liver disease occur mostly 20–30 year after infection putting a significant burden on the health care and economical system [6].

There is evidence that treatment leading to a sustained viral response (SVR) can reverse the effects of early stage fibrosis, and slow the progression of cirrhosis into decompensation or HCC [7,8]. Thus, prompt identification and management of cases is imperative for mitigation of the increasing burden of disease resulting from an aging population [9]. There is not currently a national screening strategy to identify HCV cases in Switzerland. However, the Federal Office of Public Health (FOPH) has collected and maintained a notification database for all positive tests for non-A, non-B hepatitis and HCV since 1988 [9,10]. The aim of this study is to expand on recent modeling efforts through novel scenario development to assist in the development of national strategies for HCV control. Additionally, the cost of untreated HCV was analyzed.

## Methods

### HCV disease progression model

A detailed description of the HCV disease progression model was described previously [11] and is provided in S1 Fig and S1 Table. The model was populated and calibrated using Swiss specific assumptions (S1 File, S2 Table) to forecast the future burden of HCV by stage of the disease. For consistency, the HCV population by sequelae in 2030 was used for comparison to the 2013 populations in the analyses below, unless noted otherwise.

#### Model Outcomes

Model outcomes included annual estimates of total viremic infections as well as viremic infections by disease sequelae (e.g. fibrosis, cirrhosis and HCC). Deaths attributable to background mortality and liver related mortality were also tracked annually. The forecasts were collected for each scenario described below.

#### Epidemiology of the HCV infected population

A thorough review of published literature and government reports was conducted to describe the total population and HCV prevalent population, and meetings with key opinion leaders and HCV experts in Switzerland were held to gain consensus surrounding the inputs. Some of the inputs summarized below have been described in more detail elsewhere [12].

Base (1.6%) and high (1.8%) estimates for anti-HCV prevalence were chosen from a modeling study completed in 1998 [13] with a low estimate of 0.8% [9]. The age and gender distribution of HCV infected individuals was estimated using notification data from the FOPH [14] and the genotype (G) distribution was available through the Swiss Hepatitis C Cohort Study [15]. The model only tracks viremic cases, and a viremic rate of 79.7% was chosen to adjust antibody estimates [16]. Applying the anti-HCV prevalence and viremic rate estimates to Swiss population data [17] indicates there were approximately 88,000 (45,400–102,000) viremic HCV infections in 1998.

Notification data and analysis from the FOPH suggest that 41,300 anti-HCV (32,900 viremic) infections were diagnosed as of 2013, with approximately 1,310 new anti-HCV (1,050 viremic) diagnoses occurring annually since 2011 [12,14].

The number of annual new cases was defined as the sum of acute infections progressing to chronicity plus chronic (viremic) infections entering Switzerland through immigration. The FOPH reports that 711 acute infections of HCV were declared from 2002–2011, or approximately 71 acute infections annually [18]. Since only 20–25% of acute infections are symptomatic [9] and an estimated 79.7% become chronic, there may have been as many as 283 new chronic infections occurring annually. Additionally, during 2002–2011, it was estimated that as many as 8,820 anti-HCV infections entered Switzerland through migration (immigrants minus emigrants) [19,20], or approximately 700 viremic infections annually, based on the assumption that only 79.7% of those anti-HCV+ are viremic [16]. Combined, these data suggest that up to 985 new viremic infections occur annually in Switzerland. The analysis assumed the number of new infections and re-infection will remain constant in the future.

Background mortality was estimated using the Human Mortality Database of the University of Berkeley and the mortality rates recorded by the Swiss Federal Statistics Office (FSO) [21,22]. Additionally, increased mortality among active injecting drug users (IDUs) was estimated using a standardized mortality ratio (SMR) of 5.5 for individuals between 15 and 44 years of age given the effective harm reduction programs in place in the Switzerland [23–25]. Although IDU was reported as a risk factor among 65% of all HCV infections, only 21% were estimated to be active users [26–29].

#### Healthcare cost data

For both in- and outpatients the costs generated in 2012 were obtained from the financial department at the University Hospital in Zürich. For each patient the fibrosis stage according to the Metavir staging system was obtained either from liver biopsy or estimated from Fibroscan reports. In case the fibrosis stage was obtained by either method and the results were not in agreement, the biopsy data was used. The records included a history of liver transplantation, HCC diagnosis or if the patient was being treated for the HCV infection. In addition, all in-patients hospitalized in 2012 with acute HCV (International Classification of Diseases (ICD) 10 code: B17.1) and/or chronic HCV (ICD 10 code: B18.2), and the respective Swiss Procedure Code (CHOP) codes were extracted from the hospital database. Liver transplant reimbursement costs were available through the hospital, so data obtained through in- and outpatient records were used solely to estimate the cost of subsequent years of care following a liver transplantation. The cost of antiviral therapy was excluded from this analysis. Although all costs were obtained from a single-center, a consensus was found within the group of experts that these are representative for Switzerland.

Previous liver transplant was noted in outpatient records, and was defined by an ICD 10 code of T864 (or subsets). HCC was defined as HCC in outpatient records, or an in- patient ICD 10 code of C220. Decompensated cirrhosis was defined as an in- patient ICD 10 code of R18, G92 or I85, in the absence of more advanced liver disease. Cirrhosis was defined as F4 in outpatient records or an in- patient ICD 10 code of K746, in the absence of more advanced liver disease. Fibrosis data was only available from outpatient records.

#### Healthcare cost analysis (excluding treatment)

Healthcare costs were calculated by matching in-patient and outpatient records and calculating a total cost for each patient. Patients were allocated to the most severe disease stage diagnosed and average cost was calculated across the total number of patients in the stage (Table 1). Finally, the average annual cost for patients with fibrosis and compensated cirrhosis were adjusted as follows to account for the zero costs incurred by the undiagnosed population.

**Table 1:** Estimated cost per patient per year (excluding the cost of antiviral therapy), by disease stage, in 2011 Euros.

Disease Stage	Base cost* (€)	Low Cost** (€)	High Cost*** (€)
Chronic HCV (F0)	104	21	643
F1	169	14	1,100
F2	381	71	1,681
F3	866	134	4,618
Compensated Cirrhosis	2,174	98	16,352
Decompensated Cirrhosis	16,457	4,223	30,117
Hepatocellular Carcinoma	13,567	1,983	58,246
Liver Transplant	100,174	87,796	239,110
Liver Transplant—Subseq. Yrs	15,473	193	171,851

(* Base costs were calculated as described above, ** Low costs represent the minimum cost associated with each stage, *** High costs represent the maximum cost associated with disease each stage).

Averageannualcost=Annualcost*%Diagnosed

This approach ignored the healthcare costs associated with HCV related comorbidities if the patients were not diagnosed with HCV. At the time of this analysis, cost of the new therapies was not available and treatment cost was excluded.

#### Sensitivity analysis

The uncertainty in assumptions/inputs was captured as a range (S3 Table) using a Beta-PERT distribution. Crystal Ball, an Excel add-in by Oracle was used to run Monte Carlo simulations to determine the 95% uncertainty intervals and sensitivity analysis.

### Scenario development

Under the base case scenario (current standard of care), an estimated 1,100 patients were treated annually in Switzerland [12,30]. The pool of patients eligible for treatment was determined by the number of diagnosed patients who were age and fibrosis stage eligible, with no contraindications to treatment and who were willing to accept treatment. It was assumed that all patients between 15–69 years of age who had a fibrosis score of F2 or higher on the Metavir scale were eligible for standard and triple therapy. The average SVR for this population was 61% for G1 and G4, and 70% for G2 and G3. Additionally, it was assumed that 40% of patients were either contraindicated or refused current therapies [31,32].

Scenarios were modeled with changes to SVR, medical eligibility, treatment uptake, diagnosis rates, treated patient segments and the timing of access to the new therapies.

1. Discontinuation of treatment—all therapies were discontinued in 2014 to assess the effect of current treatment standards.
2. Increased treatment uptake and efficacy to achieve 50% or 90% reduction in mortality—two scenarios were developed to achieve a 50% and 90% reduction in liver-related disease burden and mortality by 2030 treating ≥F2 patients using antivirals with a 95% efficacy rate. Additionally, scenarios were run where the fibrosis stage threshold for starting therapy was increased to ≥F3 or F4 (including decompensated cirrhosis and transplant), keeping constant the goal of 50% and 90% reductions in mortality
3. Impact of time and patient segment—the impact of time was assessed by delaying the ≥F2-90%-reduction scenario by 2 or 5 years. The impact of patient segment was assessed by restricting treatment to ≥F3 or F4 patients. For all of these scenarios, SVR increases, as well as the number of treated patients remained the same as in the ≥F2-90%-reduction scenario.
4. 90% reduction ‘hybrid’ scenario—the ≥F2-90%-reduction was modified to initially restrict treatment to F4 patients beginning in 2014. Restrictions were relaxed in 2016 (to include ≥F3) and 2018 (to include ≥F2).

## Results

### Base Case

**Fig 1:**
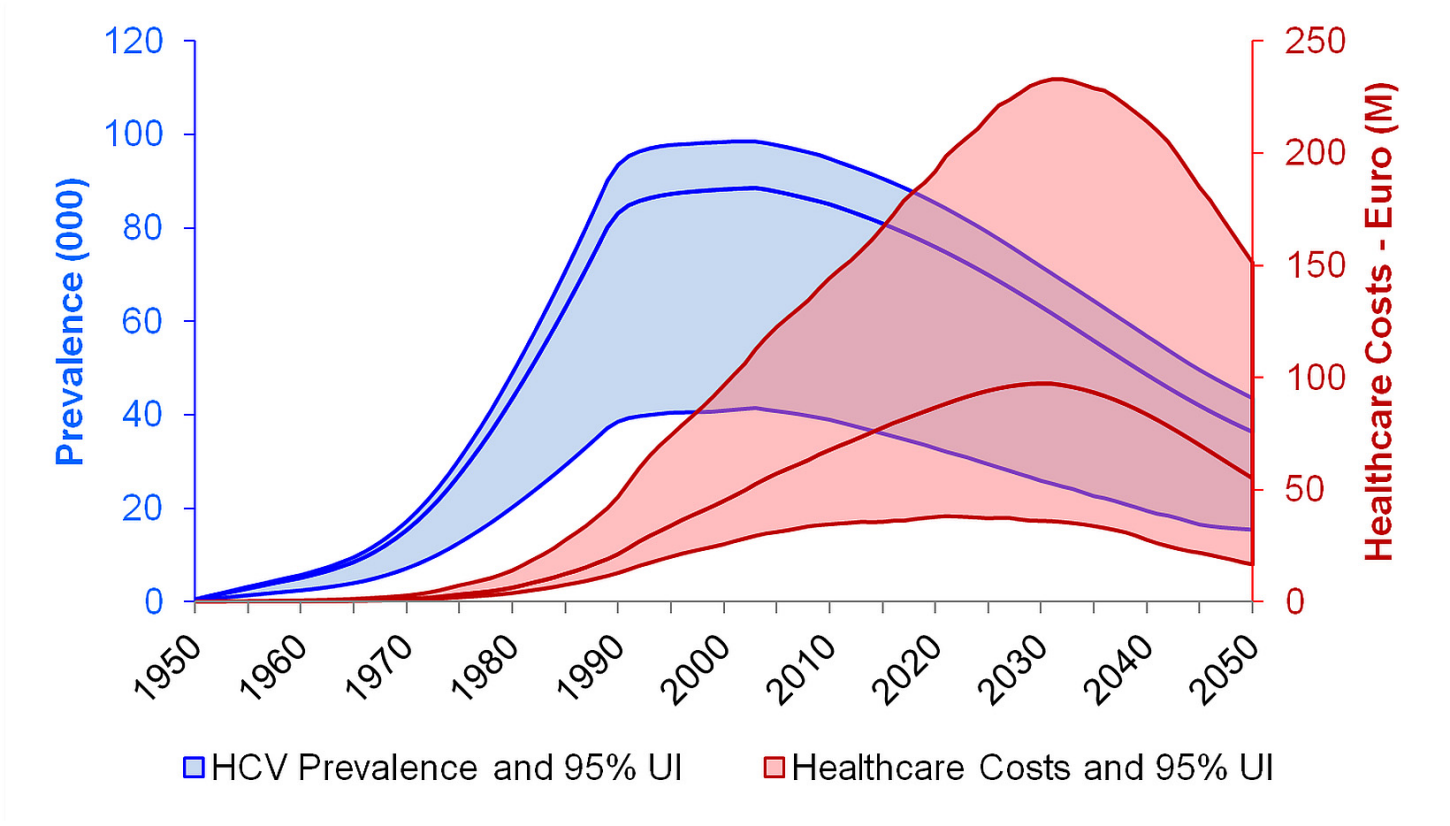
Total viremic cases (blue) and associated healthcare costs (red), by year, 1950–2050 (the upper and lower bounds represent 95% uncertainty intervals).

**Fig 2:**
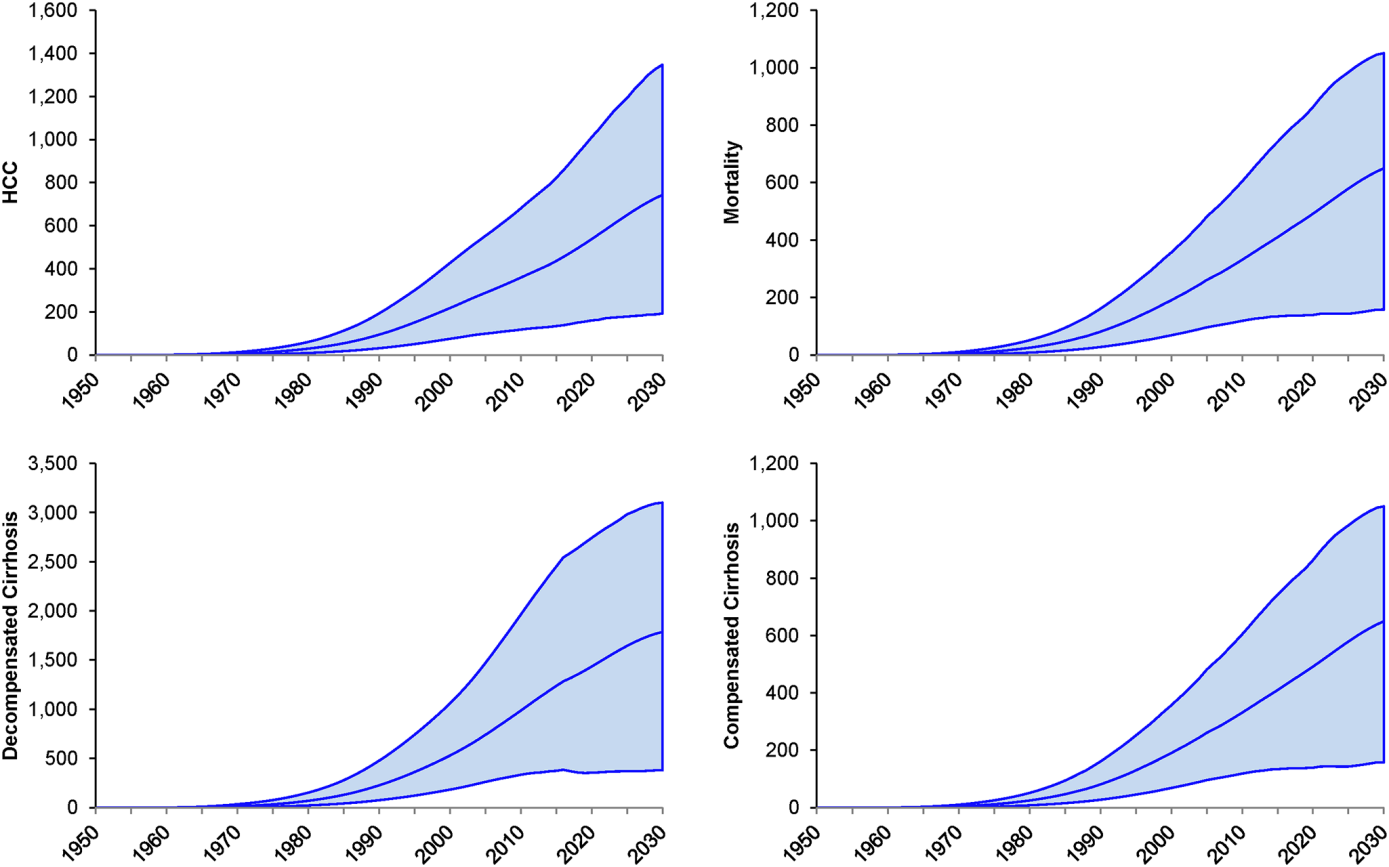
Change in disease burden, by year, 1950–2030 (the upper and lower bounds represent 95% uncertainty intervals).

Peak viremic prevalence of chronic HCV infection was reached in 2003 at 88,600 (41,500–98,500) viremic infections, and by 2013 there were 82,700 (37,200–93,400) infections (Fig 1). Although prevalence was estimated to decline to 63,200 (25,900–71,800) viremic infections in 2030, assuming that all inputs and outputs remain stable over time (including treatment uptake and efficacy) the number of individuals with advanced liver disease increased. The number of compensated cirrhosis (n = 12,700), decompensated cirrhosis (n = 1,790) and HCC (n = 745) cases were forecasted to increase 50%, 57% and 84% as compared to the 2013 populations (Fig 2). Additionally, by 2030, liver related mortality was forecasted to increase 72% (from 380 deaths to 650 deaths) as the HCV population ages (Fig 2).

The 2013 annual healthcare cost of viremic HCV (excluding antiviral treatment costs) was estimated at €74 (€36 –€157) million. The annual cost was projected to peak in 2030 at €97 (€36 –€232) million (Fig 1). Under the base case, the cumulative healthcare costs for 2013–2030 were estimated at €1,581 (€620 –€4,053) million.

The results of the sensitivity analysis are shown in S2 Fig. The analysis showed that the uncertainty in the anti-HCV prevalence is the driver of uncertainty accounting for most of the observed variability.

### Discontinuation of Treatment

**Fig 3:**
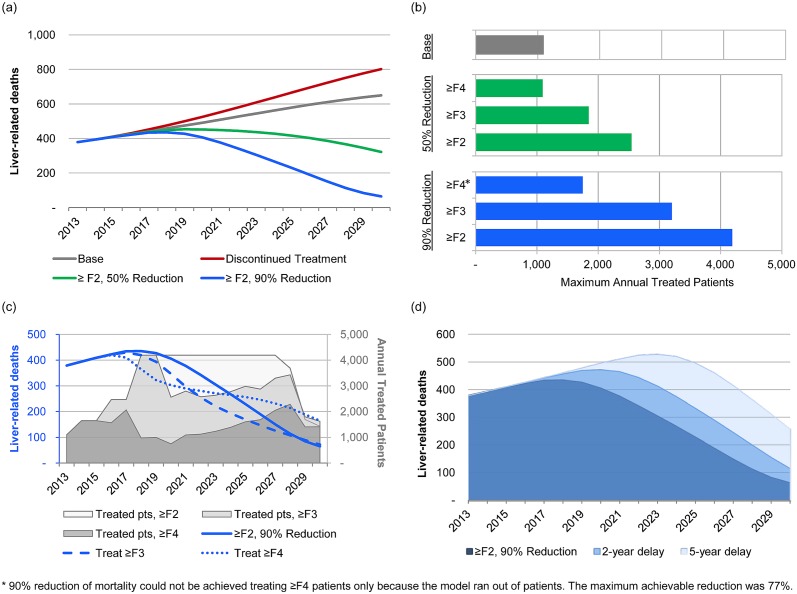
Exploratory scenario outputs, 2013–2030. (A) HCV-related liver-related deaths, by scenario; (B) Maximum number of patients treated annually to achieve 50% or 90% reduction, by METAVIR stage treated; (C) Impact of treatment restrictions (restriction to ≥F3 or F4) on a strategy to reduce liver-related mortality by 90%, and the annual number of patients treated before ‘running out of patients’; (D) Impact of 2-year and 5-year delays on a strategy to reduce liver-related mortality by 90%.

In the absence of treatment, viremic prevalence was forecasted to decrease at a slower rate than under the base case, with 73,400 viremic cases in 2030. Additionally, by 2030, the numbers of individuals with compensated cirrhosis and decompensated cirrhosis were projected to increase to 16,485 and 2,280 cases, respectively. The number of individuals with HCC, caused by HCV infection, was projected to increase to 910 cases, a 22% increase over the base case. Under this scenario, liver related mortality was projected to increase from 380 deaths in 2013 to 800 deaths in 2030, a 23% increase over the base case (Fig 3a).

Discontinuing treatment was associated with a projected €190 million per year increase in net costs from 2013–2030 as compared with the base case (cumulative cost = €1,771 million). The increase in cost was a result of the progression to more advanced disease by those who would have been cured under the base scenario. In 2030, annual costs were projected to be 24% higher than under the base case.

### Treatment uptake required to achieve 50% or 90% reduction in mortality

In order to attain 50% reduction in mortality while restricting access to ≥F2 patients, an increase in treatment of up to 2,550 patients annually by 2018, with 95% SVR therapies, would be required (Table 2, Fig 3b). This strategy increases the number of patients, with a 50% increase in the number of patients treated beginning in 2016 (1,640 total) and a 155% increase beginning in 2018 (2,550 total). The model was designed to provide a warning when an insufficient number of patients was available for treatment. In order for treatment to increase at this rate, improved screening and diagnosis (i.e. from 1,050 viremic diagnoses annually in 2013 to 2,370 viremic diagnoses annually in 2020) and the treatment of patients up to 74 years of age were necessary.

**Table 2:** Maximum annual treatment and diagnosis required for 50% and 90% reductions in HCC/Mortality, by treated stage.

Scenario		Annual Treatment		Cumulative Treatment	Annual Diagnosis
		Max	2030	2013–2030	Max
Base		1,100		19,700	1,050
50% Reduction	≥F2	2,550		39,700	2,370
	≥F3	1,850		30,000	2,370
	F4	1,095		19,700	3,580
90% Reduction	≥F2	4,190	1,430	58,000	5,370
	≥F3	3,200	1,640	46,900	6,010
	F4*	*1*,*750*	*1*,*415*	*26*,*100*	*7*,*900*
	Hybrid	4,190	1,495	57,955	5,370

* A 90% reduction in mortality could not be attained if treatment was restricted to the F4 population.

To attain 90% reduction in mortality while limiting access to ≥F2 patients, an increase in treatment of up to 4,190 patients annually by 2018, with 95% SVR therapies, would be required (Table 2, Fig 3b). This strategy increases the number of patients gradually, with a 50% increase in the number of treated patients beginning in 2014 (1,640 total), 50% increase beginning in 2016 (2,460 total), and 70% increase beginning in 2019 (4,190 total). In order for treatment to increase at this rate, improved screening and diagnosis is necessary (from 1,050 viremic diagnoses annually in 2013 to 5,370 viremic diagnoses annually in 2020) and the treatment of patients up to 79 years of age is compulsory.

Restricting therapy access to ≥F3 or F4 patients decreased the treatment rate required to reach 50% or 90% reduction (Table 2, Fig 3b), however the number of viremic patients diagnosed annually had to be increased, as shown in Table 2, to have sufficient number of patients to treat. Furthermore, 90% reduction could not be attained if treatment was restricted to the F4 population.

### Scenario analyses around the ≥F2-90% reduction scenario

Restricting treatment to ≥F3 patients resulted in a rapid initial decrease in liver-related mortality, however liver related deaths in 2030 were very similar to those under the ≥F2-90%-reduction scenario (≥F3–70 deaths in 2030, ≥F2–65 deaths in 2030) (Fig 3c, Table 3). Treating 4,190 ≥F3 patients annually was only sustainable until 2020, after which time the model predicted that only 2,500–3,400 patients were available to treat annually. Since fewer patients were treated cumulatively, the number of viremic infections increased 35%, as compared with the ≥F2 scenario.

**Table 3:** Model outputs by scenario, and scenario analysis surrounding ≥F2, 90% reduction in HCC/Mortality scenario.

	**Model Outputs by Scenario**	**HCC**			**Mortality**			**Prevalence**		**Decomp Cirrhosis**			**Comp Cirrhosis**		
		**2013**		**2030**	**2013**		**2030**	**2013**	**2030**	**2013**		**2030**	**2013**		**2030**
	**Base**	405		745	380		650	82,700	63,200	1,140		1,790	8,520		12,700
		**HCC**			**Mortality**			**Prevalence**		**Decomp Cirrhosis**			**Comp Cirrhosis**		
	**Scenario**	**Peak (Year)**		**2030**	**Peak (Year)**		**2030**		**2030**	**Peak (Year)**		**2030**	**Peak (Year)**		**2030**
Modeled Scenarios	Discontinued Treatment	910	(2030)	910	800	(2030)	800		73,400	2,280	(2030)	2,280	16,480	(2030)	16,485
	≥F2, 50% Reduction in Mortality	485	(2020)	350	455	(2019)	320		42,600	1,280	(2017)	730	9,250	(2017)	4,990
	≥F2, 90% Reduction in Mortality	455	(2018)	70	435	(2018)	65		29,400	1,270	(2016)	110	8,820	(2015)	825
	Hybrid, 90% Reduction in Mortality	420	(2015)	75	420	(2019)	70		29,500	1,230	(2015)	120	8,670	(2014)	895
Scenario Analysis, Max 4,190 tx / yr	≥F2, 90% Reduction														
	2- year delay	500	(2020)	100	470	(2020)	115		30,000	1,360	(2018)	170	9,450	(2017)	1,235
	5-year delay	565	(2023)	230	525	(2019)	260		35,700	1,500	(2021)	450	10,420	(2020)	3,010
	Treat ≥F3	440	(2017)	65	430	(2019)	70		40,000	1,260	(2016)	115	8,670	(2014)	1,140
	Treat F4	420	(2015)	150	420	(2019)	165		57,500	1,230	(2015)	250	8,670	(2014)	3,880

Restrict to F4—Restricting treatment to F4 patients resulted in a very rapid initial decrease in liver related deaths followed by a plateau (Fig 3c). By 2030, there were 165 liver related deaths annually, 154% more than under the ≥F2 scenario (65 cases as shown in Table 3). The viremic prevalence in 2030 was 57,500—a 95% increase from the ≥F2 scenario (29,400).

Delay by 2 years—Delaying the ≥F2-90%-reduction scenario’s implementation by two years resulted in a 77% increase in liver related mortality by 2030 (Fig 3d, Table 3).

Delay by 5 years—Delaying the implementation by five years resulted in a 300% increase in liver related mortality by 2030 (Fig 3d, Table 3).

### Hybrid scenario to reduce mortality by 90%

**Fig 4:**
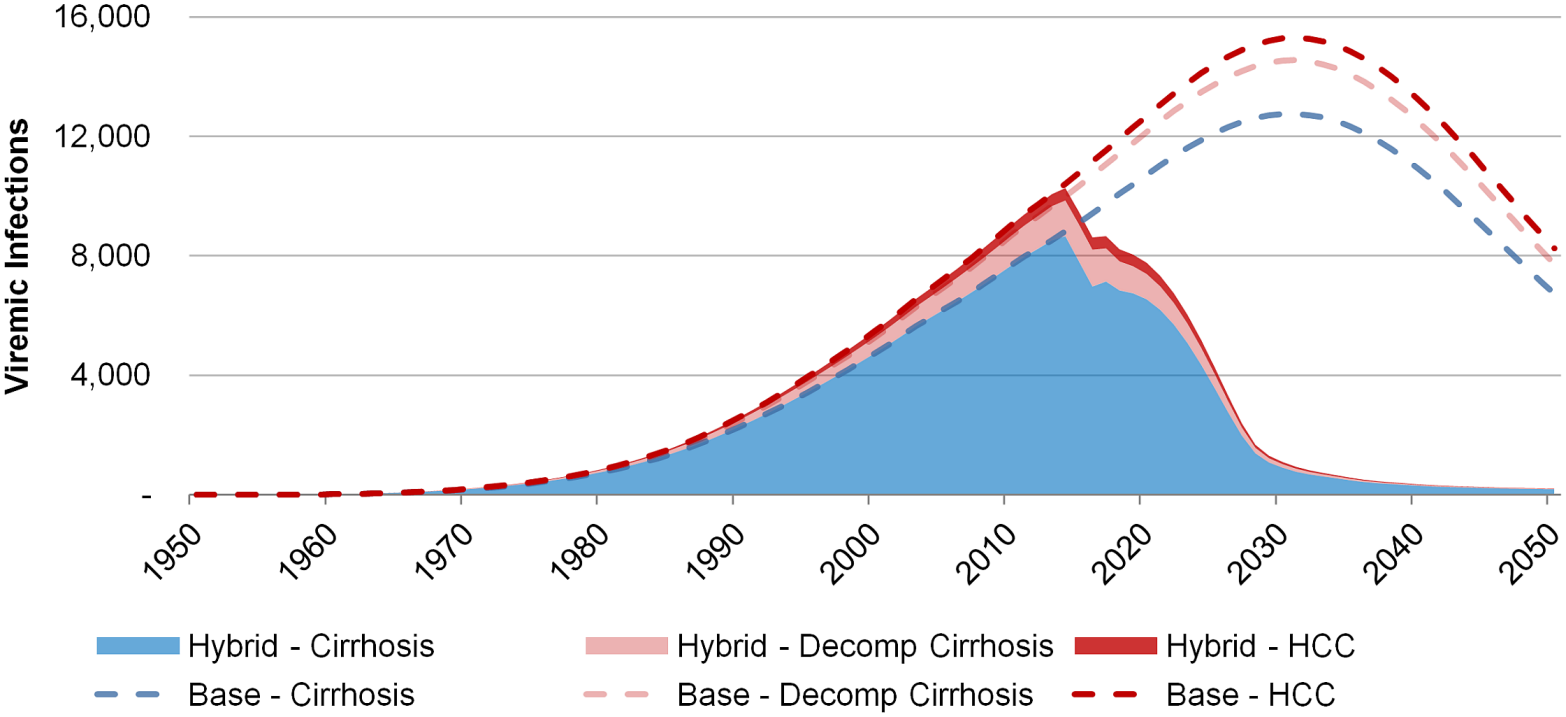
Projected base and hybrid scenario results for cirrhosis, decompensated cirrhosis and HCC, 1950–2050.

The hybrid approach to reduce mortality by 90% resulted in both a rapid initial decrease in liver-related deaths and allowed for sustained treatment of 4,190 patients annually until 2028. Thus, the viremic prevalence in 2030 was similar to that of the ≥F2-90%-reduction scenario, with 29,500 cases (compared with 29,400) (Table 3). The impact of the hybrid approach on reductions in compensated and decompensated cirrhosis, as well as HCC can be seen in Fig 4.

## Discussion

Despite a decrease in the rate of new infections in Switzerland, HCV poses a considerable public health threat caused by secondary liver related morbidity and mortality of those already infected. This is further complicated by the fact that less than 50% of the infected population is aware of their infection [9].

With today’s treatment efficacy and uptake rates, the number of cases of HCV induced cirrhosis, decompensated cirrhosis and HCC is expected to steadily increase until 2030. It is important to note, however, that prevention (of disease or mortality) is an intangible outcome and recognition of current efforts is worth acknowledging. The analysis in which treatment was discontinued showed a 23% increase in mortality compared with current treatment, suggesting the importance of, at minimum, maintaining the current standard of care to prevent increased morbidity and mortality.

A key insight of this analysis was identification of factors that drive a reduction in HCV morbidity, mortality and total infections. A reduction in end stage liver diseases (ESLD) and liver related deaths (LRD) is achievable by focusing treatment on patients with high fibrosis (≥F3 or F4); however, a significant reduction in the total number of infections cannot be achieved unless treatment is expanded to patients in the early stages of fibrosis. The trade-off to achieve a reduction in total infections is the requirement for a higher number of patients to be treated to achieve the same level of reduction in ESLD and LRD by 2030. A significant reduction in the total number of infection is not achievable under the current treatment paradigm.

If managing the drug costs while minimizing liver related deaths, is the primary goal, then focusing on patients with late stage of fibrosis would achieve a significant reduction in HCV morbidity and mortality while treating fewer patients. Once this population has been depleted, treatment can be expanded to patients with a lower fibrosis (≥F2 before ≥F1 before ≥F0) as demonstrated by the hybrid strategy. This strategy ensures that the patients who need treatment most are given a priority while managing the treatment cost and the increased demand on the healthcare system.

However, the analysis also showed that focusing treatment exclusively on F4 patients was not as effective in reducing LRD. This is driven by three factors: only those who are diagnosed can be treated, not all individuals with fibrosis score of F4 are diagnosed and individuals who are F4 tend to be older. Aside from the fact that decompensated cirrhotics cannot be treated with any combination therapy containing interferon, individuals who are F4 and not diagnosed will continue to progress and age. Once they pass age of 74, the analysis considers them ineligible for treatment. These individuals are ineligible for treatment independent of their future diagnosis status. In addition, studies have shown that some cirrhotic patients will continue to progress to ESLD after achieving SVR [8]. Thus, some portion of the F4 patients who are diagnosed, treated, and achieve SVR will continue to progress. Treating patients after they are cirrhotic is less effective than preventing them from becoming cirrhotic. Expansion of treatment to ≥F3 captured these individuals while they were younger than 74 years old and prevented disease progression.

This highlights another important observation—the impact of treatment to prevent disease progression. As shown in Fig 3C, the ≥F3 strategy was more effective in reducing LRD after 2021 as compared to the F4 strategy. This was because patients who achieved SVR were prevented from progressing to cirrhosis and subsequently ESLD. Those F3 cases who are undiagnosed will still have a chance to be diagnosed when they reach F4 thus improving the odds of capturing and treating them before they reach ESLD.

A strategy to reduce HCV prevalence by 90% in Switzerland must involve treatment regardless of liver disease stage. This approach would require a significant expansion of the current treatment capacity, possibly through appropriately trained mid-level providers and/or primary care physicians [33], i.e. according to a task shifting strategy already effectively tested for HIV infection [34,35] and recently proposed for HCV [36]. Once well tolerated and easy to administer therapies become available, a test-and-treat approach (where an HCV diagnosis is followed by a treatment offer) may be possible. The availability of such simplified regimens, effective for all patients independently of their baseline and on-treatment features, may further raise the trend of treating HCV as an infectious disease.

Increased detection and diagnosis of viremic cases is a primary requirement for all strategies aiming to significantly impact HCV disease burden. Therefore, enhancing detection rates in Switzerland has high priority in order to control the HCV epidemic. Birth cohort screening among individuals born between 1945 and 1965 has proven effective in the United States (US) [37], and a similar approach could be considered for Switzerland. The Swiss HCV epidemic is younger than that of the US, with 70% of the viremic population born between 1950 and 1975. Screening programs targeting this population are estimated to have the greatest success in identifying new cases.

Given the higher SVR of the new therapies, delays in access lead to slower reductions in mortality, morbidity and total infections between 2013 and 2030 (Table 3). A 2 and 5 years delay in the ≥F2 strategies lead to 80% and 300% higher liver related deaths in years 2030.

There were a number of limitations in this study that impact the accuracy of our base projections. Sensitivity analysis identified prevalence as the largest driver of uncertainty in the Swiss model—accounting for 88% of the variability. A wide range around prevalence was purposefully chosen to account for the variability in currently cited estimates; however, this underscores the need for a reliable, population based survey. Though not captured in the sensitivity analysis, additional uncertainty surrounds the notification data. Mandatory reporting of HCV in Switzerland is based on a passive surveillance system and is therefore subject to underreporting. However, this underreporting is limited by the fact that all cases are laboratory confirmed and that many laboratories have automated routines to report positive cases. Additionally, many patients have been tested several times, which increases the likelihood of cases being reported. Finally, there is uncertainty surrounding the source of infection, as 40% of reports cited “unknown” or missing routes of transmission. Percent of transmission due to IDU and transfusion were therefore calculated only among cases with a known exposure.

Finally, the analysis focused on ESLD and mortality and the impact of treatment as prevention was not considered. The latter would result in a faster drop in the total number of HCV infections by reducing HCV transmission. The impact of treatment as prevention on ESLD and LRD would be minimal in the time frame considered (2013–2030) since new infected individuals will take time to progress to ESLD.

In order to improve detection and treatment of HCV, Switzerland needs to coordinate its efforts nationwide. While the strategies mentioned in this paper are contingent upon the availability of new potent therapies with higher cure rates and improved treatment experience, the current standard of care shows a reduction in disease burden as compared to the modeled effects of discontinuing all treatment. Further modeling analyses are needed to evaluate the cost effectiveness of these aforementioned strategies, including the cost of medication. The analysis presented here highlights the need to develop timely strategies to reduce the effects of chronic HCV disease in Switzerland.

## Supporting Information

S1 Fig The flow of the HCV disease progession model. (TIF)

S2 Fig Tornado diagram highlighting the key drivers for the 2013 viremic prevalence estimate, with percent of variation explained by each parameter. (TIF)

S1 File General Population Assumptions. (DOCX)

S1 Table HCV Disease Progression Rates. (DOC)

S2 Table Base HCV(+) population assumptions. (DOC)

S3 Table Parameters Included in Sensitivity and Uncertainty Analysis. (DOC)
