# Supplementary figures and images for: Modeling the Health and Economic Burden of Hepatitis C Virus in Switzerland
Source: PLoS One. 2015 Jun 24;10(6):e0125214. doi: 10.1371/journal.pone.0125214 (PMC4480969; doi:10.1371/journal.pone.0125214)

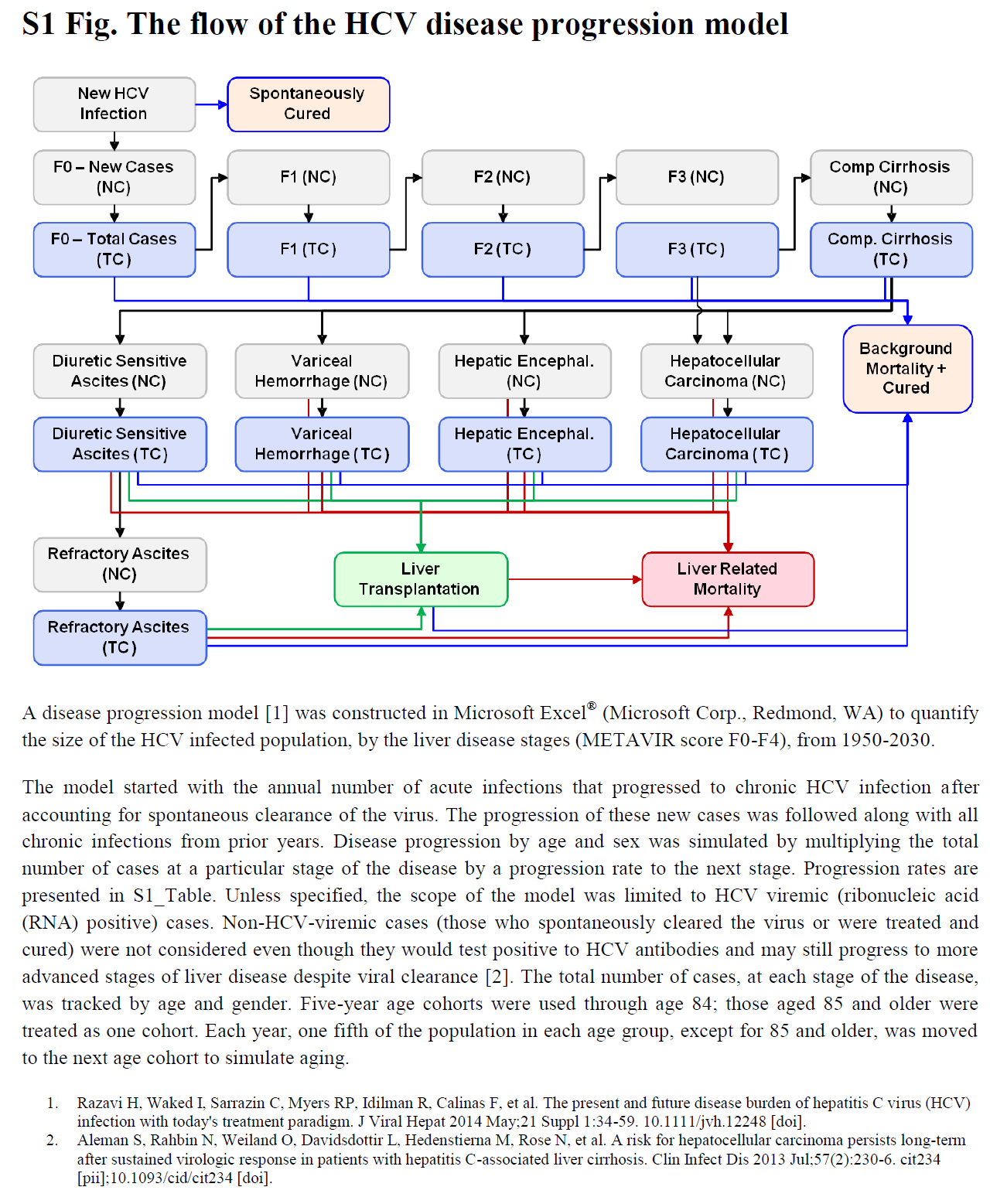

Supplement: S1 Fig — (TIF) [file pone.0125214.s001.tif]

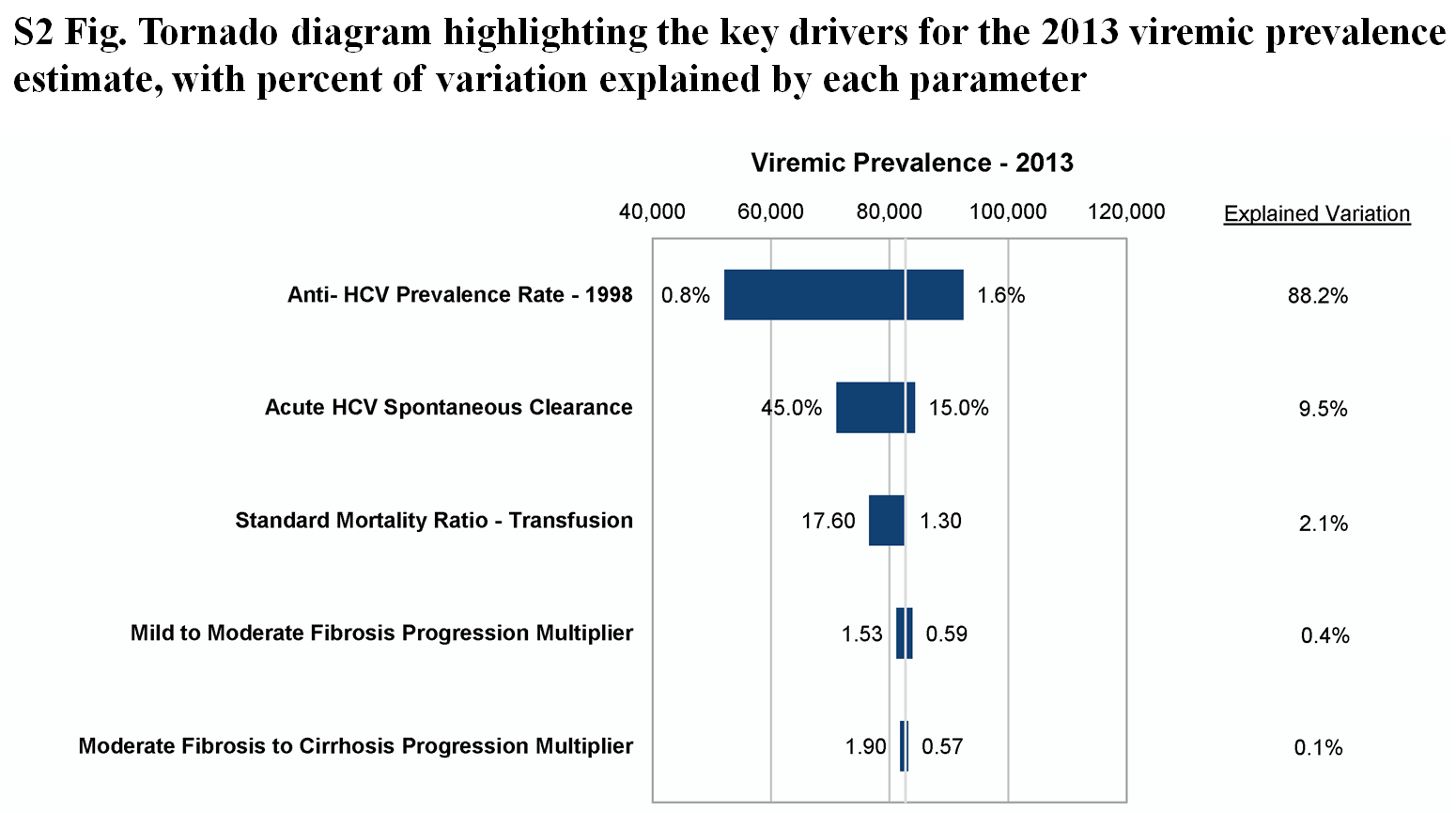

Supplement: S2 Fig — (TIF) [file pone.0125214.s002.tif]
