# Supplementary material for: Modeling the Health and Economic Burden of Hepatitis C Virus in Switzerland
Source: PLoS One. 2015 Jun 24;10(6):e0125214. doi: 10.1371/journal.pone.0125214 (PMC4480969; doi:10.1371/journal.pone.0125214)
Supplement: S1 File — (DOCX) [file pone.0125214.s003.docx]

## S1 File. General Population Assumptions

### Swiss General Population

Population data were obtained through the Swiss Federal Statistics Office (FSO) and the United Nations (UN) Population Database [1;2]. As data through FSO were only available from 1971-2010, calculations were necessary to adjust the UN data to match local data. Calculations were performed as follows:

- Before 1971—population estimates were calculated using a 5 year average ratio of UN and FSO data from 1971-1975. This ratio was then applied to UN data prior to 1971
- 1971-2010 FSO data were used
- After 2010-- population estimates were calculated using a 5 year average ratio of UN and FSO data from 2006-2010 UN and FSO data. This ratio was then applied to UN data after 2010

### Swiss General Mortality Rates

Mortality rates were obtained through the Swiss Federal Statistics Office (FSO) and the Max Planck Institute for Demographic Research’s Human Mortality Database (HMD) [3;4]. Similar to population data, mortality data through FSO were only available from 1971-2010, so calculations were necessary to adjust the UN data to match local data. Calculations were performed as follows:

- Before 1971—mortality estimates were calculated using a 5 year average ratio from 1971-1975 HMD and FSO data, applied to HMD data
- 1971-2010 FSO data were used
- After 2010-- mortality estimates were calculated using a 5 year average ratio from 2006-2010 HMD and FSO data, applied to HMD data

Reference List

1. Swiss Federal Statistics Office. Age Statistics, 1971-2010: Age, demographic component, nationality, gender. STAT-TAB: The interactive statistical database.2013 [cited:Jun 17 2013] Available from: URL: http://www.pxweb.bfs.admin.ch/Dialog/statfile.asp?lang=1

2. United Nations.Dept.of Economic and Social Affairs.Population Division (2011). World population prospects: The 2010 revision, Volume I: Comprehensive tables. ST/ESA/SER.A/313.2011 Available from: URL: http://esa.un.org/wpp/Documentation/pdf/WPP2010_Volume-I_Comprehensive-Tables.pdf

3. University of California,B, Mack Planck Institute for Demographic Research. Human Mortality Database.Jun 14 2013 [cited:Feb 1 2013] Available from: URL: www.mortality.org

4. Swiss Federal Statistics Office. Fatalities by institutional divisions, gender, nationality, marital status and age group, 1971-2010. STAT-TAB: The interactive statistical database.2013 [cited:Jun 17 2013] Available from: URL: http://www.pxweb.bfs.admin.ch/Dialog/statfile.asp?lang=1
