# Supplementary material for: Modeling the Health and Economic Burden of Hepatitis C Virus in Switzerland
Source: PLoS One. 2015 Jun 24;10(6):e0125214. doi: 10.1371/journal.pone.0125214 (PMC4480969; doi:10.1371/journal.pone.0125214)
Supplement: S1 Table — (DOC) [file pone.0125214.s004.doc]

**S1 Table. HCV Disease Progression Rates**

# Reported Progression rates:

| **Disease Progression** | **Reported Progression Rates** | **Source** |
| --- | --- | --- |
| Acute HCV Spontaneous Clearance | 18.0% (15.0-45.0%) | [1-3] |
| Diur Sens Ascites to Diur Refractory Ascites | 6.7% (4.0-9.4%) | [1-9] |
| Diur Sens Ascites to Liver Related Death | 11.0% (7.7-14.3%) | [1-9] |
| Variceal Hem. to Liver Related Death (Yr 1) | 40.0% (33.4-46.6%) | [1-9] |
| Variceal Hem. to Liver Related Death (Sub Yrs) | 13.0% (8.5-17.5%) | [1-9] |
| Hepatic Enceph. to Liver Related Death (Yr 1) | 68.0% (65.9-70.1%) | [1-9] |
| Hepatic Enceph. to Liver Related Death (Sub Yrs) | 40.0% (37.8-42.2%) | [1-9] |
| Diur Refractory Ascites to Liver Related Death | 33.0% (28.0-38.0%) | [1-9] |
| HCC to Liver Related Death (Yr 1) | 70.7% (43-77.0%) | [8;10] |
| HCC to Liver Related Death (Sub Yrs) | 16.2% (11-23.0%) | [10] |
| Liver Transplant to Liver Related Death (Yr 1) | 33.1-10.7% (SD 2.8-0.4%) | [11;12] |
| Liver Transplant to Liver Related Death (Sub Yrs) | 3.9-4.8% (SD 7.6%-1.0) | [11;12] |

Source: Razavi H, Waked I, Sarrazin C, et al. The present and future disease burden of hepatitis C virus (HCV) infection with today's treatment paradigm. J Viral Hepat 2014; 21

Suppl 1: 34-59.

# Progression rates used in the model, back-calculated and adjusted by age and gender:

| **Age Cohorts** | 0-4 | 5-9 | 10-14 | 15-19 | 20-24 | 25-29 | 30-34 | 35-39 | 40-44 | 45-49 | 50-54 | 55-59 | 60-64 | 65-69 | 70-74 | 75-79 | 80-84 | 85+ |
| --- | --- | --- | --- | --- | --- | --- | --- | --- | --- | --- | --- | --- | --- | --- | --- | --- | --- | --- |
| **Back-calculated progression rates - males** | | | | | | | | | | | | | | | | | | |
| F0 to F1 | 5.3% | 5.3% | 6.4% | 6.4% | 5.2% | 5.2% | 3.8% | 3.8% | 13.9% | 13.9% | 17.1% | 17.1% | 19.4% | 19.4% | 21.8% | 21.8% | 17.9% | 17.9% |
| F1 to F2 | 3.8% | 3.8% | 4.7% | 4.7% | 3.8% | 3.8% | 2.7% | 2.7% | 10.1% | 10.1% | 12.4% | 12.4% | 14.1% | 14.1% | 15.8% | 15.8% | 13.0% | 13.0% |
| F2 to F3 | 5.4% | 5.4% | 6.6% | 6.6% | 5.3% | 5.3% | 3.9% | 3.9% | 14.3% | 14.3% | 17.5% | 17.5% | 19.9% | 19.9% | 22.4% | 22.4% | 18.3% | 18.3% |
| F3 to Cirrhosis | 0.0% | 0.0% | 0.8% | 0.8% | 2.5% | 2.5% | 5.7% | 5.7% | 8.8% | 8.8% | 4.8% | 4.8% | 9.9% | 9.9% | 19.1% | 19.1% | 19.1% | 19.1% |
| F3 to HCC | 0.0% | 0.0% | 0.0% | 0.0% | 0.0% | 0.0% | 0.0% | 0.0% | 0.1% | 0.1% | 0.1% | 0.1% | 0.2% | 0.2% | 0.3% | 0.3% | 0.3% | 0.3% |
| Cirrhosis to HCC | 0.3% | 0.3% | 0.3% | 0.3% | 0.3% | 0.3% | 0.5% | 0.5% | 0.9% | 0.9% | 1.4% | 1.4% | 2.4% | 2.4% | 3.9% | 3.9% | 3.9% | 3.9% |
| **Back-calculated progression rates - females** | | | | | | | | | | | | | | | | | | |
| F0 to F1 | 4.4% | 4.4% | 5.4% | 5.4% | 4.3% | 4.3% | 3.1% | 3.1% | 11.6% | 11.6% | 14.3% | 14.3% | 16.2% | 16.2% | 18.2% | 18.2% | 14.9% | 14.9% |
| F1 to F2 | 3.2% | 3.2% | 3.9% | 3.9% | 3.1% | 3.1% | 2.3% | 2.3% | 8.4% | 8.4% | 10.4% | 10.4% | 11.7% | 11.7% | 13.2% | 13.2% | 10.8% | 10.8% |
| F2 to F3 | 4.5% | 4.5% | 5.5% | 5.5% | 4.4% | 4.4% | 3.2% | 3.2% | 11.9% | 11.9% | 14.6% | 14.6% | 16.6% | 16.6% | 18.6% | 18.6% | 15.3% | 15.3% |
| F3 to Cirrhosis | 0.0% | 0.0% | 0.6% | 0.6% | 2.1% | 2.1% | 4.7% | 4.7% | 7.4% | 7.4% | 4.0% | 4.0% | 8.3% | 8.3% | 15.9% | 15.9% | 15.9% | 15.9% |
| F3 to HCC | 0.0% | 0.0% | 0.0% | 0.0% | 0.0% | 0.0% | 0.0% | 0.0% | 0.0% | 0.0% | 0.1% | 0.1% | 0.1% | 0.1% | 0.2% | 0.2% | 0.2% | 0.2% |
| Cirrhosis to HCC | 0.3% | 0.3% | 0.3% | 0.3% | 0.3% | 0.3% | 0.4% | 0.4% | 0.7% | 0.7% | 1.2% | 1.2% | 2.0% | 2.0% | 3.3% | 3.3% | 3.3% | 3.3% |

Source: Razavi H, Waked I, Sarrazin C, et al. The present and future disease burden of hepatitis C virus (HCV) infection with today's treatment paradigm. J Viral Hepat 2014; 21

Suppl 1: 34-59.

Reference List

1. Thomas DL, Seeff LB. Natural history of hepatitis C. Clin Liver Dis 2005 Aug;9(3):383-98. S1089-3261(05)00035-8 [pii];10.1016/j.cld.2005.05.003 [doi].
2. Alter MJ, Margolis HS, Krawczynski K, Judson FN, Mares A, Alexander WJ, et al. The natural history of community-acquired hepatitis C in the United States. The Sentinel Counties Chronic non-A, non-B Hepatitis Study Team. N Engl J Med 1992 Dec 31;327(27):1899-905.
3. Villano SA, Vlahov D, Nelson KE, Cohn S, Thomas DL. Persistence of viremia and the importance of long-term follow-up after acute hepatitis C infection. Hepatology 1999 Mar;29(3):908-14. S0270913999001378 [pii];10.1002/hep.510290311 [doi].
4. Razavi H, Elkhoury AC, Elbasha E, Estes C, Pasini K, Poynard T, et al. Chronic hepatitis C virus (HCV) disease burden and cost in the United States. Hepatology 2013 Jun;57(6):2164-70. 10.1002/hep.26218 [doi].
5. Deuffic-Burban S, Deltenre P, Buti M, Stroffolini T, Parkes J, Muhlberger N, et al. Predicted effects of treatment for HCV infection vary among European countries. Gastroenterology 2012 Oct;143(4):974-85. S0016-5085(12)01151-1 [pii];10.1053/j.gastro.2012.05.054 [doi].
6. Thein HH, Yi Q, Dore GJ, Krahn MD. Estimation of stage-specific fibrosis progression rates in chronic hepatitis C virus infection: A meta-analysis and meta-regression. Hepatology 2008 Aug;48(2):418-31. 10.1002/hep.22375 [doi].
7. Bennett WG, Inoue Y, Beck JR, Wong JB, Pauker SG, Davis GL. Estimates of the cost- effectiveness of a single course of interferon-alpha 2b in patients with histologically mild chronic hepatitis C. Ann Intern Med 1997 Nov 15;127(10):855-65.
8. Bernfort L, Sennfalt K, Reichard O. Cost-effectiveness of peginterferon alfa-2b in combination with ribavirin as initial treatment for chronic hepatitis C in Sweden. Scand J Infect Dis 2006;38(6-7):497-505. PK71701726364401 [pii];10.1080/00365540500532803 [doi].
9. Younossi ZM, Singer ME, McHutchison JG, Shermock KM. Cost effectiveness of interferon alpha2b combined with ribavirin for the treatment of chronic hepatitis C. Hepatology 1999 Nov;30(5):1318-24. S0270913999004954 [pii];10.1002/hep.510300518 [doi].
10. Ries L, Young G, Keel G, Eisner M, Lin Y, Horner M. SEER survival monograph: Cancer survival among adults: U.S. SEER program, 1988-2001, patient and tumor characteristics. [NIH Pub. No. 07-6215]. 2007. Bethesda, MD, National Cancer Institute, SEER Program.

Ref Type: Serial (Book,Monograph)

1. Organ Procurement and Transplantation Network (OPTN). National Data. Health Resources and Services Administration, U.S. Department of Health & Human Services; 2013.
2. Organ Procurement and Transplantation Network (OPTN). 2009 OPTN/SRTR annual report 1999-2008: Table 9.15a. Unadjusted patient survival by year of transplant at 3 months, 1 year, 3 years, 5 years and 10 years, deceased donor liver transplants. 2009 Annual report of the U.S.Organ Procurement and Transplantation Network and the Scientific Registry of Transplant

Recipients: Transplant data 1999-2008 . 2009. Rockville, MD, U.S. Department of Health and Human Services, Health Resources and Services Administration, Healthcare Systems Bureau, Division of Transplantation. 10-19-2012. Available from: <http://www.ustransplant.org/annual_reports/current/915a_li.htm>
