# Supplementary material for: Modeling the Health and Economic Burden of Hepatitis C Virus in Switzerland
Source: PLoS One. 2015 Jun 24;10(6):e0125214. doi: 10.1371/journal.pone.0125214 (PMC4480969; doi:10.1371/journal.pone.0125214)
Supplement: S2 Table — (DOC) [file pone.0125214.s005.doc]

**S2 Table Base HCV(+) population assumptions**

| **Parameter** | **Year of Estimate** | **Value (range)** | **Source** |
| --- | --- | --- | --- |
| **HCV Antibody Positive (000)** | | | |
| Prevalence | 1998 | 1.6% (0.8% - 1.8%) | [1–3] |
| Viremic Rate |  | 79.7% | [4] |
| **Genotypes** | 1994, 2012 |  | [5] |
| 1a |  | 26% |  |
| 1b |  | 26% |  |
| 1 Other |  | - |  |
| 1 |  | 52% |  |
| 2 |  | 9% |  |
| 3 |  | 29% |  |
| 4 |  | 10% |  |
| 5 |  | 0% |  |
| 6 |  | 0% |  |
| **Diagnosed (Viremic)** | | | |
| Total Cases | 2012 | 32,900 | [6] |
| Annual Newly Diagnosed | 2012 | 1,100 | [3] |
| **Treated & Cured** | | | |
| Annual Number Treated | 2011 | 1,100 | [7] |
| SVR G1 | 2013 | 61% | [8] |
| SVR G2 | 2013 | 70% | [8] |
| SVR G3 | 2013 | 70% | [8] |
| SVR G4 | 2013 | 61% | [8] |
| **Liver Transplants** | | | |
| Total Number of Liver  Transplants | 2011 | 109 | [9] |
| % due to HCV |  | 21% | See below |

Liver transplant percent attributable to HCV

| **2009 2010** | **2011** | **2012** | **Total** |
| --- | --- | --- | --- |
| Liver Transplants 102 100 | 109 | 100 | 945 |
| HCV-related Transplants (over 4 years) |  |  | 87 |
| Percent attributable to HCV |  |  | 21.2% |

Reference List

1. Sagmeister M, Renner EL, Mullhaupt B, Wong JB. Simulation of hepatitis C based on a mandatory reporting system. Eur J Gastroenterol Hepatol 2002 Jan;14(1):25-34.
2. Fretz R, Negro F, Bruggmann P, Lavanchy D, De Gottardi A, Pache I, et al. Hepatitis B and C in Switzerland-healthcare provider initiated testing for chronic hepatitis B and C infection. Swiss Med Wkly 2013;143:0. 10.4414/smw.2013.13793 [doi];smw-13793 [pii].
3. Swiss Federal Office of Public Health. Number of hepatitis C cases reported in Switzerland between 1988 and 2012 by year of birth (mandatory notification of laboratory confirmed cases): FOPH/ID/EPI/RIC. 2013.
4. Armstrong GL, Wasley A, Simard EP, McQuillan GM, Kuhnert WL, Alter MJ. The prevalence of hepatitis C virus infection in the United States, 1999 through 2002. Ann Intern Med 2006 May 16;144(10):705-14.144/10/705 [pii].
5. The Swiss Hepatitis C Cohort Study. Swiss hepatitis C cohort study report-March 31, 2012.2012 [cited:Mar 7 2013] Available from: <http://www.swisshcv.ch/pdf/Report_SCCS_March_31_2012.pdf>
6. Swiss Federal Office of Public Health. Estimated number of notified hepatitis C cases still alive in 2012: FOPH/ID/EPI/RIC. 2013.
7. IMS Health. IMS Health MIDAS Data. IMS Health Jan 1 2013 Available from: URL: [http://www.imshealth.com/portal/site/ims/menuitem.edb2b81823f67dab41d84b903208c22a/?vgn](http://www.imshealth.com/portal/site/ims/menuitem.edb2b81823f67dab41d84b903208c22a/?vgnextoid=4475e3de7e390310VgnVCM1000007f8c2ca2RCRD)  [extoid=4475e3de7e390310VgnVCM1000007f8c2ca2RCRD](http://www.imshealth.com/portal/site/ims/menuitem.edb2b81823f67dab41d84b903208c22a/?vgnextoid=4475e3de7e390310VgnVCM1000007f8c2ca2RCRD)
8. EASL Clinical Practice Guidelines: Management of hepatitis C virus infection. J Hepatol 55: 245-264. S0168-8278(11)00209-1 [pii];10.1016/j.jhep.2011.02.023 [doi].
9. Swiss National Foundation for Organ Donation and Transplantation. Swiss transplant annual report 2012. 2013. Bern, Switzerland, Stampfli Publikationen AG.
