# Supplementary material for: Modeling the Health and Economic Burden of Hepatitis C Virus in Switzerland
Source: PLoS One. 2015 Jun 24;10(6):e0125214. doi: 10.1371/journal.pone.0125214 (PMC4480969; doi:10.1371/journal.pone.0125214)
Supplement: S3 Table — (DOC) [file pone.0125214.s006.doc]

**S3 Table. Parameters Included in Sensitivity and Uncertainty Analysis**

| **Parameter** | **Base** | **Low** | **High** | **Source** | **Distribution** |
| --- | --- | --- | --- | --- | --- |
| ***Disease Progression Rates*** | | | | | |
| Acute HCV Spontaneous Clearance | 18.0% | 15.0% | 45.0% | [1–3] | Beta-PERT |
| Mild to moderate fibrosis^1^ | 1.00 | 0.59 | 1.53 | *[4] | Triangular |
| Moderate fibrosis to cirrhosis^1^ | 1.00 | 0.57 | 1.90 | *[4] | Triangular |
| Compensated to decompensated cirrhosis^1^ | 1.00 | 0.70 | 1.36 | *[4] | Triangular |
| Cirrhosis to HCC^1^ | 1.00 | 0.74 | 1.32 | *[4] | Triangular |
| Diur Sens Ascites to Diur Refrac Ascites | 6.7% | 4.0% | 9.4% | [1–3,5–  10] | Beta-PERT |
| Diur Sens Ascites to Liver Rel. Death | 11.0% | 7.7% | 14.3% | [1–3,5–  10] | Beta-PERT |
| Diur Refrac Ascites to Liver Rel. Death | 33.0% | 28.0% | 38.0% | [1–3,5–  10] | Beta-PERT |
| Variceal Hem. to Liver Rel. Death (Yr 1) | 40.0% | 33.4% | 46.6% | [1–3,5–  10] | Beta-PERT |
| Variceal Hem. to Liver R. Death (Sub Yrs) | 13.0% | 8.5% | 17.5% | [1–3,5–  10] | Beta-PERT |
| Hepatic Enceph. to Liver Rel. Death (Yr 1) | 68.0% | 65.9% | 70.1% | [1–3,5–  10] | Beta-PERT |
| Hepatic Enceph. to Liver R. Death (Sub Yrs) | 40.0% | 37.8% | 42.2% | [1–3,5–  10] | Beta-PERT |
| HCC to Liver Rel. Death (Yr 1) | 70.7% | 43.0% | 77.0% | [9,11] | Beta-PERT |
| HCC to Liver R. Death (Sub Yrs) | 16.2% | 11.0% | 23.0% | [11] | Beta-PERT |
| Liver Transplant 1988-2010 Rate | 2.2% | 2.1% | 3.2% | Calculated | Beta-PERT |
| Liver Transplant 2011-2030 Rate | 3.0% | 2.9% | 4.5% | Calculated | Beta-PERT |
| ***Input variables*** | | | | | |
| Anti-HCV Prevalence Rate - 1998 | 1.2% | 0.8% | 1.6% | [12–14] | Beta-PERT |
| Percent of HCV(+) with IDU risk | 61% | 34% | 67% | [15] | Beta-PERT |
| Standard Mortality Ratio - IDU | 5.5 | 4.0 | 7.0 | [16–18] | Beta-PERT |
| Standard Mortality Ratio - Transfusion | 2.1 | 1.3 | 17.6 | [19] | Beta-PERT |
| ***Cost^2^*** | | | | | |
| Chronic HCV (F0) Annual Cost | 769 | 153 | 4,760 | [20] | Beta-PERT |
| F1 Annual Cost | 784 | 64 | 5,087 | [20] | Beta-PERT |
| F2 Annual Cost | 1,081 | 202 | 4,772 | [20] | Beta-PERT |
| F3 Annual Cost | 1,897 | 293 | 10,119 | [20] | Beta-PERT |
| Cirrhosis Annual Cost | 3,393 | 153 | 25,526 | [20] | Beta-PERT |
| Decompensated Cirrhosis Annual Cost | 20,553 | 5,273 | 37,611 | [20] | Beta-PERT |
| Hepatocellular Carcinoma Annual Cost | 16,944 | 2,477 | 72,741 | [20] | Beta-PERT |
| Liver Transplant Annual Cost (Yr 1) | 125,102 | 109,644 | 298,614 | [20] | Beta-PERT |
| Liver Transplant Annual Cost (Sub Yrs) | 19,323 | 241 | 214,616 | [20] | Beta-PERT |
| ^1^Multipliers are applied to base transition probabilities, by age and gender, during uncertainty analysis  ^2^Cost inputs for uncertainty analysis are presented prior to adjusting for diagnosis rate  ^*^Derived from progression rates published by Harris et al 2013 [4] | | | | | |

Reference List

1. Thomas DL, Seeff LB. Natural history of hepatitis C. Clin Liver Dis 2005 Aug;9(3):383-98. S1089-3261(05)00035-8 [pii];10.1016/j.cld.2005.05.003 [doi].
2. Alter MJ, Margolis HS, Krawczynski K, Judson FN, Mares A, Alexander WJ, Hu PY, Miller JK, Gerber MA, Sampliner RE. The natural history of community-acquired hepatitis C in the United States. The Sentinel Counties Chronic non-A, non-B Hepatitis Study Team. N Engl J Med 1992 Dec 31;327(27):1899-905.
3. Villano SA, Vlahov D, Nelson KE, Cohn S, Thomas DL. Persistence of viremia and the importance of long-term follow-up after acute hepatitis C infection. Hepatology 1999 Mar;29(3):908-14. S0270913999001378 [pii];10.1002/hep.510290311 [doi].
4. Harris RJ, Thomas B, Griffiths J, Costella A, Chapman R, Ramsay M, et al. Increased uptake and new therapies are needed to avert rising hepatitis C-related end stage liver disease in England: Modelling the predicted impact of treatment under different scenarios. J Hepatol 2014 Sep;61(3):530-7. S0168-8278(14)00318-3 [pii];10.1016/j.jhep.2014.05.008 [doi].
5. Razavi H, Elkhoury AC, Elbasha E, Estes C, Pasini K, Poynard T, Kumar R. Chronic hepatitis C virus (HCV) disease burden and cost in the United States. Hepatology 2013 Jun;57(6):2164- 70.10.1002/hep.26218 [doi].
6. Deuffic-Burban S, Deltenre P, Buti M, Stroffolini T, Parkes J, Muhlberger N, et al. Predicted effects of treatment for HCV infection vary among European countries. Gastroenterology 2012 Oct;143(4):974-85. S0016-5085(12)01151-1 [pii];10.1053/j.gastro.2012.05.054 [doi].
7. Thein HH, Yi Q, Dore GJ, Krahn MD. Estimation of stage-specific fibrosis progression rates in chronic hepatitis C virus infection: A meta-analysis and meta-regression. Hepatology 2008 Aug;48(2):418-31. 10.1002/hep.22375 [doi].
8. Bennett WG, Inoue Y, Beck JR, Wong JB, Pauker SG, Davis GL. Estimates of the cost- effectiveness of a single course of interferon-alpha 2b in patients with histologically mild chronic hepatitis C. Ann Intern Med 1997 Nov 15;127(10):855-65.
9. Bernfort L, Sennfalt K, Reichard O. Cost-effectiveness of peginterferon alfa-2b in combination with ribavirin as initial treatment for chronic hepatitis C in Sweden. Scand J Infect Dis 2006;38(6-7):497-505. PK71701726364401 [pii];10.1080/00365540500532803 [doi].
10. Younossi ZM, Singer ME, McHutchison JG, Shermock KM. Cost effectiveness of interferon alpha2b combined with ribavirin for the treatment of chronic hepatitis C. Hepatology 1999 Nov;30(5):1318-24. S0270913999004954 [pii];10.1002/hep.510300518 [doi].
11. Ries L, Young G, Keel G, Eisner M, Lin Y, Horner M. SEER survival monograph: Cancer survival among adults: U.S. SEER program, 1988-2001, patient and tumor characteristics. [NIH Pub. No. 07-6215]. 2007. Bethesda, MD, National Cancer Institute, SEER Program. Ref Type: Serial (Book,Monograph).
12. Sagmeister M, Renner EL, Mullhaupt B, Wong JB. Simulation of hepatitis C based on a mandatory reporting system. Eur J Gastroenterol Hepatol 2002 Jan;14(1):25-34.
13. Fretz R, Negro F, Bruggmann P, Lavanchy D, De Gottardi A, Pache I, et al. Hepatitis B and C in Switzerland-healthcare provider initiated testing for chronic hepatitis B and C infection. Swiss Med Wkly 2013;143:0. 10.4414/smw.2013.13793 [doi];smw-13793 [pii].
14. Swiss Federal Office of Public Health. Number of hepatitis C cases reported in Switzerland between 1988 and 2012 by year of birth (mandatory notification of laboratory confirmed cases): FOPH/ID/EPI/RIC. 2013.
15. Swiss Federal Office of Public Health. Source of infection among mandatory notified cases of hepatitis C, Switzerland 1993-2011: FOPH/MT/EPI/RIC. 2012.
16. Hickman M, Hope V, Coleman B, Parry J, Telfer M, Twigger J, et al. Assessing IDU prevalence and health consequences (HCV, overdose and drug-related mortality) in a primary care trust: implications for public health action. J Public Health (Oxf) 2009 Sep;31(3):374-82. fdp067 [pii];10.1093/pubmed/fdp067 [doi].
17. Degenhardt L, Hall W, Warner-Smith M. Using cohort studies to estimate mortality among injecting drug users that is not attributable to AIDS. Sex Transm Infect 2006 Jun;82 Suppl 3:iii56-iii63. 82/suppl_3/iii56 [pii];10.1136/sti.2005.019273 [doi].
18. Mathers BM, Degenhardt L, Bucello C, Lemon J, Wiessing L, Hickman M. Mortality among people who inject drugs: a systematic review and meta-analysis. Bull World Health Organ 2013 Feb 1;91(2):102-23. 10.2471/BLT.12.108282 [doi];BLT.12.108282 [pii].
19. Kamper-Jorgensen M, Ahlgren M, Rostgaard K, Melbye M, Edgren G, Nyren O, et al. Survival after blood transfusion. Transfusion 2008 Dec;48(12):2577-84. TRF01881 [pii];10.1111/j.1537- 2995.2008.01881.x [doi].
20. Mullhaupt B (2013) Analysis of inpatient and outpatient costs associated with HCV.
